# Unveiling the Hidden Burden: An Audit Examining Whether National Institute for Health and Care Excellence (NICE) Guidelines Are Followed in the Management of Depression in Primary Care

**DOI:** 10.1192/bjo.2026.11553

**Published:** 2026-06-30

**Authors:** Aileen Anthonipillai

**Affiliations:** City St George’s University of London, London, United Kingdom

## Abstract

**Aims:**

**Background:**

Depression is estimated to affect approximately 10% of patients in primary care. Timely follow-up is crucial for monitoring symptom progression, assessing suicide risk, and evaluating treatment response. NICE (2022) recommend review within 2–4 weeks of diagnosis, or within 1 week if suicide risk is identified, while the Quality and Outcomes Framework (QOF) provide a broader window of 10–56 days. Despite the integral role of primary care in the management of depression, adherence to these standards varies widely across UK practices, putting patients at risk of clinical deterioration and suicide.

Assessing compliance with NICE guidelines for depression can identify gaps and guide strategies to improve patient safety and clinical outcomes. This audit therefore evaluated adherence to NICE guidelines in a South London GP practice, with a focus on review timing and documentation of suicide risk assessment.

**Aims:**

1. To determine the proportion of patients with newly diagnosed depression who received a follow-up review within 2–4 weeks

2. To assess documentation of suicidal risk assessment and subsequent follow-up.

**Methods:**

A retrospective audit of EMIS records identified all patients diagnosed with “Depression” or “Mixed depressive and anxiety disorder” from January and May 2024 (n=70). Fourteen patients were excluded as the 4-week review window had not yet elapsed, leaving 56 patients for analysis. Data were collected across nine parameters, including review timing, suicide risk documentation, treatment initiation, comorbidities, and referrals to specialist or psychological services.

**Results:**

The sample included 56 patients (59% female, mean age of 39.6 years). A notable 59% of patients had no documented review, with nearly half having recorded comorbidities. Only 41% had a documented review (n=23), with just 25% (n=14) being reviewed within the NICE recommended 2–4-week timeframe, while 39% met the QOF target of 10-56 days. Suicide risk assessment was documented in 91% of cases; however, 27% of patients reporting suicidal ideation had no documented referral to specialist or psychological services.

**Conclusion:**

Adherence to NICE-recommended follow up for depression in primary care was low, with significant gaps in referral for patients with suicidal ideation, posing concerns regarding patient safety. Targeted strategies such as staff training on guideline compliance, automated follow-up prompts within EMIS, and integration of mental health reviews within multimorbidity consultations may improve compliance and support safer depression management. Ongoing monitoring and larger multi-site audits are needed to guide quality improvement in depression management on a broader scale.

